# Tectorigenin: A Review of Its Sources, Pharmacology, Toxicity, and Pharmacokinetics

**DOI:** 10.3390/molecules28155904

**Published:** 2023-08-05

**Authors:** Juan Rong, Fei Fu, Chenxia Han, Yaling Wu, Qing Xia, Dan Du

**Affiliations:** 1 West China Centre of Excellence for Pancreatitis, Institute of Integrated Traditional Chinese and Western Medicine, Sichuan Provincial Pancreatitis Centre and West China-Liverpool Biomedical Research Centre, West China Hospital, Sichuan University, Chengdu 610041, China; rongjuan812@gmail.com (J.R.); hanchenxia@scu.edu.cn (C.H.); 2 Advanced Mass Spectrometry Center, Research Core Facility, Frontiers Science Center for Disease-Related Molecular Network, West China Hospital, Sichuan University, Chengdu 610041, China; fufei9940@wchscu.cn (F.F.); 2022224070024@stu.scu.edu.cn (Y.W.); 3 Proteomics-Metabolomics Platform, Research Core Facility, West China-Washington Mitochondria and Metabolism Centre, Institutes for Systems Genetics, West China Hospital, Sichuan University, Chengdu 610041, China

**Keywords:** tectorigenin, sources, extraction and synthesis, pharmacology, toxicology, pharmacokinetics, delivery strategy

## Abstract

Tectorigenin is a well-known natural flavonoid aglycone and an active component that exists in numerous plants. Growing evidence suggests that tectorigenin has multiple pharmacological effects, such as anticancer, antidiabetic, hepatoprotective, anti-inflammatory, antioxidative, antimicrobial, cardioprotective, and neuroprotective. These pharmacological properties provide the basis for the treatment of many kinds of illnesses, including several types of cancer, diabetes, hepatic fibrosis, osteoarthritis, Alzheimer’s disease, etc. The purpose of this paper is to provide a comprehensive summary and review of the sources, extraction and synthesis, pharmacological effects, toxicity, pharmacokinetics, and delivery strategy aspects of tectorigenin. Tectorigenin may exert certain cytotoxicity, which is related to the administration time and concentration. Pharmacokinetic studies have demonstrated that the main metabolic pathways in rats for tectorigenin are glucuronidation, sulfation, demethylation and methoxylation, but that it exhibits poor bioavailability. From our perspective, further research on tectorigenin should cover: exploring the pharmacological targets and mechanisms of action; finding an appropriate concentration to balance pharmacological effects and toxicity; attempting diversified delivery strategies to improve the bioavailability; and structural modification to obtain tectorigenin derivatives with higher pharmacological activity.

## 1. Introduction

It is well known that natural products or botanicals are valuable resources in the treatment of dozens of diseases. Up until now, natural products remain an important basis for the development of emerging therapeutic agents [1]. Newman and Cragg (2020) showed that between 1981 and 2019, natural products, natural product derivatives, and botanical drugs accounted for 33.5% of all newly approved drugs [2]. Flavonoids are secondary metabolites of plants that function as signaling molecules, antioxidants, and detoxifying agents, and protect plants against various biotic and abiotic threats [3]. Flavonoids belong to polyphenol groups, and have been found to exert anti-inflammatory [4], antioxidative [5], antibacterial [6], antidiabetic [7], neuroprotective [8], anticancer, and other activities [9,10,11,12]. Therefore, they have the potential to be used in several fields, including medicine [13], nutraceuticals as dietary supplements [14], as well as the food and cosmetic industries as biopreservatives for their antibacterial activity together with antioxidant potential [15,16].

Tectorigenin is an isoflavone that exists in numerous plant resources, and it is especially abundant in Belamcandae Rhizoma and Puerariae flos [17,18], which both have the effects of clearing away heat, removing toxic substances, relieving sore throat, and reducing swelling [19,20]. As one of the most important active ingredients, the pharmacological effects of tectorigenin have been demonstrated by an increasing number of investigators [21,22,23,24]. Due to its extensive pharmacological activities, including anticancer, antioxidation, hepatoprotection, anti-inflammation, etc., tectorigenin has received a large amount of attention [21,22,23,24]. This paper mainly reviews the research on the sources, the extraction and synthesis, the pharmacology, the toxicity, the pharmacokinetics, and the delivery strategy of tectorigenin in order to assist researchers to better understand and make use of it.

## 2. Sources of Tectorigenin

**Figure 1:**
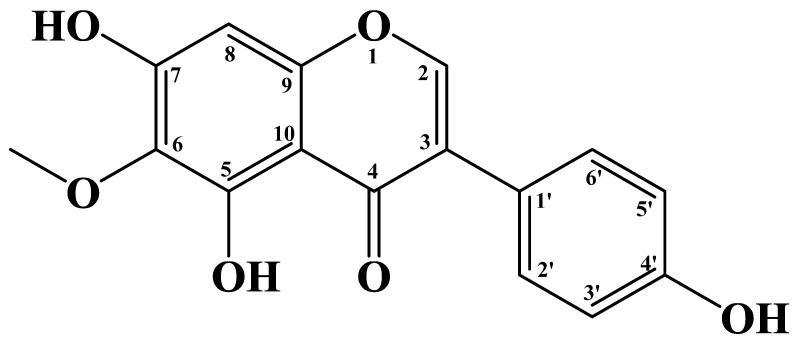
Chemical structure of tectorigenin.

Tectorigenin, a methoxy isoflavone with three hydroxyl groups, is chemically noted as 5,7-dihydroxy-3-(4-hydroxyphenyl)-6-methoxy-4H-chromen-4-one (Figure 1). As a bioactive compound, tectorigenin is found in several plant species, most notably in the rhizomes of *Belamcanda chinensis* (L.) DC., a traditional Chinese medicine (TCM) named Belamcandae Rhizoma [25,26]. The Iris family is the major source of tectorigenin, and many Iridaceous plants, such as *Iris spuria* L. (Calizona) [27], *Iris tectorum* Maxim [28], *Iris japonica* Thunb. [29,30], *Iris dichotoma* Pall. [31], *Iris germanica* L. [32], *Iris unguicularis* Poiret [33], *Iris loczyi* Kan. [33], *Iris kashmiriana* Baker [34], *Iris crocea* Jacq. ex R. C. Foster [35,36], *Iris ensata* Thunb. [36], *Iris germanica* L. [37], *Iris hungarica* Waldst. et Kit. [38], *Iris confusa* Sealy [37], and *Iris pseudacorus* L. [37], contain abundant tectorigenin, so tectorigenin is also known as iris flavone [39]. Tectorigenin also exists in some leguminous plants, such as the flowers of *Pueraria lobata* (Willd.) Ohwi [40,41], *Pueraria thomsonii* Benth. [18,40], and *Pueraria thunbergiana* Benth. [42], which are also TCM called Puerariae Flos. Tectorigenin was isolated from the leaves of *Dalbergia odorifera* T. Chen [43], the heartwood of *Dalbergia parviflora* Roxb. [44], and the roots of *Euchresta formosana* (Hayata) Ohwi as well [45]. Moreover, some plants in other families also contain tectorigenin, including *Codonopsis pilosula* (Franch.) Nannf. (Campanulaceae) [46], *Morus alba* L. (Moraceae) [46], *Viola hondoensis* W. Becker et H. Boissieu. (Violaceae) [47], and *Eleocharis dulcis* (Burm. f.) Trin. ex Hensch. (Cyperaceae) [48]. The plant sources of tectorigenin are shown in Table 1.

## 3. Extraction, Isolation, and Synthesis of Tectorigenin

The extraction and isolation of tectorigenin from plants with a high tectorigenin content have been widely performed in practical industrial production. For instance, tectorigenin can be prepared on a large scale from Belamcandae Rhizoma and Puerariae Flos [17,40,49]. The extraction methods of tectorigenin from dried slices or powders of raw materials include impregnation [50], ultrasonic extraction [17], reflux extraction [51], etc. Methanol, ethanol, and their aqueous solution are the most commonly used extraction solvents [40,52,53]. In addition, supercritical fluid extraction (with liquid CO_2_) was also used in some studies [54]. The crude extracts can be obtained by partitioning with non-polar organic solvents, such as ethyl acetate [55], chloroform [17], methylene chloride [49], etc. Moreover, repeated thin-layer chromatography and column chromatography were often performed for further isolation and purification [51]. In most cases, silica gel [56], and octadecyl silane were used as stationary phases [57]. Joung et al. [17] used the method of 70% ethanol ultrasound, partition with chloroform, and purification by silica gel column chromatography, and the yield of tectorigenin was 0.387 mg/g from 1 kg Belamcandae Rhizoma. To improve extraction efficiency, Li et al. [57] first developed an ionic-liquid-based ultrasound-assisted extraction method applied to the isolation of active compounds from *Pueraria thomsonii*, and an extraction yield of about 0.15 mg/g tectorigenin was obtained from 500 g raw material by countercurrent chromatography and semipreparative liquid chromatography. High levels of glycoside forms of tectorigenin, such as tectoridin, also exist in Belamcandae Rhizoma and Puerariae Flos, so the yield of tectorigenin can be increased by acid hydrolysis during extraction [58]. Kim et al. acidized the extract of the flower of *Pueraria thunbergiana* with H_2_SO_4_ to a final concentration of 1% (*w*/*v*), and the tectorigenin content increased from 17.10 µmol/g before acid hydrolysis to 49.58 µmol/g [59].

**Figure 2:**
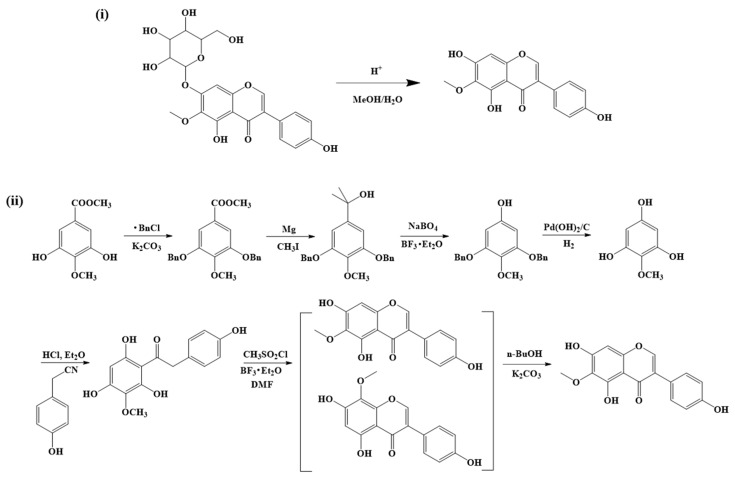
Synthesis of tectorigenin. (**i**) The synthesis of tectorigenin by hydrolyzing tectoridin; (**ii**) The de novo synthesis of tectorigenin from3-methoxy-methyl gallate.

In addition to being isolated from plants, there are two approaches to obtaining synthetic tectorigenin (Figure 2): (i) It can be produced by hydrolyzing tectoridin with 3% HCl in MeOH/H_2_O (1:1, *v*/*v*) at 85 ℃ under reflux for 4 h in MeOH/H_2_O with a yield of around 37.5% [60,61], and (ii) the de novo synthesis of tectorigenin for the first time, which was reported by Kagal et al. (1956) [62], although the yield was very low. Subsequent attempts to synthesize tectorigenin failed because of the generation of a mixture of tectorigenin and ψ-tectorigenin [63,64]. In 1965, Varady [65] pointed out that ψ-tectorigenin could be catalyzed into tectorigenin through the isomerisation of electrons rearranged under alkaline conditions with alcoholic potash and alcoholic potassium ethylate by reflux. In this context, Xiao et al. [66,67] proposed a route to synthesize tectorigenin from 3-methoxy-methyl gallate.

## 4. Pharmacological Insights of Tectorigenin

**Figure 3:**
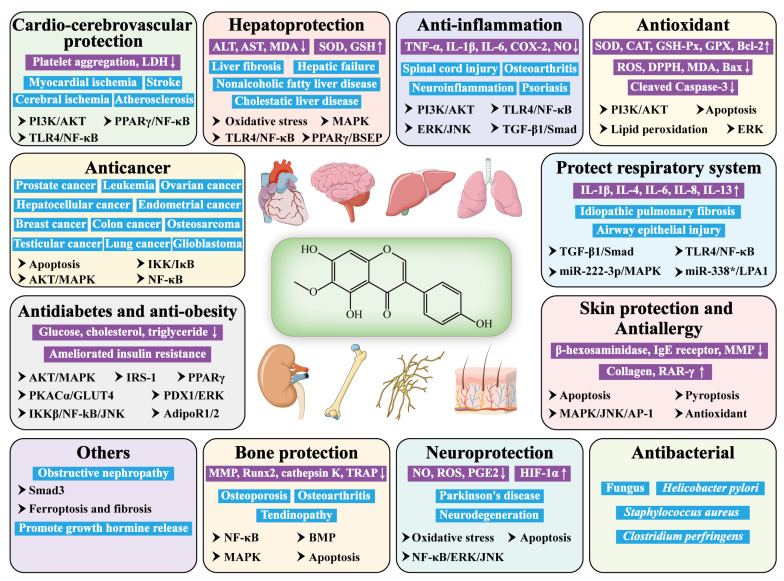
Pharmacological activity and relative mechanism of tectorigenin. (“↑” means up-regulation; “↓” means down-regulation).

As a ubiquitous plant isoflavone, tectorigenin has been widely reported to be effective in many areas, such as anticancer, anti-diabetes, hepatoprotection, anti-inflammation, antioxidation, antimicrobial, etc. (Figure 3 and Table 2). It seems to exert its pharmacological effects via modulating the signaling pathways, including PPARγ/NF-κB, PI3K/AKT, TLR4/NF-κB, IKKβ/NF-κB/JNK, ERK/JNK, MAPK/JNK/AP-1, AKT/MAPK, and TGF-β1/Smad.

### 4.1. Anticancer Effects

#### 4.1.1. Prostatic Cancer

Several epidemiological studies support the role of phytoestrogens, nonsteroidal plant-derived compounds with estrogenic activity, in reducing cancer risk [68]. In the prostate, phytoestrogens could bind to estrogen receptor β (ERβ), which might be closely related to the prevention of prostate cancer progression [68,69]. Isoflavones were reported to exhibit estrogenic effects [70,71,72]. A study revealed that tectorigenin, as a phytoestrogen, could reverse the abnormal expression of some key factors that lead to prostate cancer malignancy [73]. Actually, tectorigenin has been proven to be a selective estrogen-receptor modulator that could bind to estrogen receptors ERα and Erβ, with a higher affinity for Erβ, and prevent the proliferation of some hormone-dependent prostate cancer cells [73,74]. Morrissey et al. (2004) [75] reported the positive effects of tectorigenin alone or combined with bicalutamide on a range of prostate epithelial cells in vitro, showing that tectorigenin caused cell cycle G1 arrest. In another study, tectorigenin extracted from the flowers of *Puerariae thomsonii* was found to possess the highest anti-proliferation activity against prostate cancer cells (IC_50_ = 0.08 μM) [53]. Stettner et al. [69] also reported that tectorigenin treatment with LNCaP prostate cancer cells up-regulated ERβ, resulting in antiproliferative effects.

#### 4.1.2. Breast Cancer

Breast cancer is a major life-threatening malignancy that affects women all around the world [76]. MCF-7, MDA-MB-231, and T-47D are three commonly used breast cancer cells as models for breast tumors [77]. Zeng et al. (2018) [78] found that tectorigenin suppressed MCF-7 and MDA-MB-231 cell proliferation both in a dose- and time-dependent manner. The mechanisms of tectorigenin on human breast cancer cell apoptosis and metastasis might owe to the downregulation of protein kinase B (AKT)/mitogen-activated protein kinase (MAPK) signaling and upregulation of the expression of the caspase family. However, two earlier reports showed an opposite phenomenon: tectorigenin could stimulate the growth of MCF-7 and T-47D [44,55]. One explanation for this discrepancy is the difference in the number of cells and the concentration of tectorigenin [78]. Therefore, future studies in more diverse cell lines and animals are needed to confirm the protective effect against breast cancer of this molecule as well as its underlying mechanisms.

#### 4.1.3. Ovarian Cancer

Ovarian cancer is another common gynecologic malignancy with a high mortality rate [79,80]. New therapeutic agents for ovarian cancer are urgently needed. It was reported that many plant-derived drugs and their derivatives induce apoptosis in ovarian cancer cell lines [81]. In paclitaxel-resistant ovarian cancer cells, tectorigenin heightened the growth-inhibitory activity of paclitaxel through downregulating the AKT/IκB kinase (IKK)/inhibitor of NF-κB (IκB)/noncanonical nuclear factor-κB (NF-κB) signaling pathway [82]. To be specific, tectorigenin combined with paclitaxel inhibited the NF-κB nuclear translocation and phosphorylation of IκB and IKK by activating caspases-3/8/9 and AKT, and downregulated the expression of NF-κB-dependent genes, thereby promoting synergistic apoptosis [82]. Another study investigated the anti-ovarian cancer effect of the methanol extract of Puerariae Flos and found that the extract presented a good anti-proliferation effect against the human ovarian cancer cell line A2780. Among the active compounds, the IC_50_ of tectorigenin against A2780 cells was 48.67 ± 0.31 μM [41].

#### 4.1.4. Lung Cancer

There are also several studies on the effect of tectorigenin on lung cancer. The ethyl acetate extract of Chinese water chestnut peel, which contains tectorigenin at levels of 12.41 mg/g, showed good inhibitory activities on human alveolar adenocarcinoma cell line A549 (IC_50_ = 776.12 μg/mL) [48]. Tectorigenin (30 mg/kg) isolated from Belamcandae Rhizoma was administered subcutaneously to mice transplanted with Lewis lung carcinoma, and the inhibition ratio on tumor volume reached 30.8% [83]. In general, tumors try to escape from the host immune system and contrive to benefit from infiltrating immune cells by altering immune cell function, thereby creating a pro-inflammatory microenvironment that is conducive to tumor progression and metastasis [34]. It was reported by Amin et al. [34] that tectorigenin could restrain the pro-inflammatory response of monocytes induced by lung cancer cells, repressing the secretion of pro-inflammatory cytokines, TNF-α and IL-6. Nevertheless, the mechanism needs to be further elucidated.

#### 4.1.5. Other Cancer

It has been shown by Jiang et al. [84] that tectorigenin could reduce the vitality of HepG2 (a human hepatocellular carcinoma cell line) in a time- and concentration-dependent manner via a mitochondrial-mediated pathway to induce apoptosis. Moreover, tectorigenin restrained the proliferation of Saos2 and U2OS, which are two osteosarcoma cell lines, and it dramatically inhibited the migration and invasion of osteosarcoma cells [21]. A similar study showed that tectorigenin inhibited tumor necrosis factor-α (TNF-α) via NF-κB inhibition, thereby decreasing CXCL10 overproduction to hinder the invasion of Caco-2, which is a human colon cancer cell line [85]. In another experiment, tectorigenin could not only induce human promyelocytic leukemia HL-60 cells differentiation into granulocytes and monocytes/macrophages but also cause intracellular apoptotic variations of DNA [86]. A point of view was put forward that the 5-hydroxyl group of tectorigenin was valuable for its cytotoxic activities, which may be instructive to us [86]. Moreover, tectorigenin lowered the expression of the stem cell factors POU5F1 and NANOG, and inhibited the proliferation of malignant testicular germ cell tumor (TGCT) cells [87]. A genomic hybridization microarrays analysis displayed that over 20% of the microarray genes, including telomeres, microdeletions, oncogenes and tumor suppressor genes, were aberrated in endometrial cancer cells treated with tectorigenin [88].

### 4.2. Antidiabetic and Anti-Obesity Effects

Diabetes is a metabolic disorder characterized by hyperglycemia that has become a global epidemic [89]. Tectorigenin was reported to effectively decrease the serum glucose level of rats in streptozotocin-induced or high-fat and high-sucrose diet models [42,90,91,92], and it was reported as a potential antidiabetic agent exhibiting potent inhibitory activity on aldose reductase in rat lenses [47,93]. In vitro, tectorigenin inhibited glucotoxicity- and lipotoxicity-induced oxidative stress, apoptosis, and endoplasmic reticulum stress in islet cells. The mechanism was that tectorigenin regulated the expression of pancreas/duodenum homeobox protein 1 (PDX1) and extracellular signal-regulated kinase (ERK) [92]. Endothelial dysfunction is frequently seen in diabetic patients [94,95]. Tectorigenin was demonstrated to alleviate diabetic nephropathy, which was attributed to its protective effect on injured endothelial cells by inhibiting inflammation and lipotoxicity, and by restoring insulin sensitivity [96]. Mechanistically, the pharmacological properties of tectorigenin were related to the adiponectin receptor 1/2 (AdipoR1/2)-mediated activation of adenosine monophosphate-activated protein kinase (AMPK) and peroxisome proliferator-activated receptor (PPAR) pathways [96]. Similarly, tectorigenin exerted positive regulation of insulin action in palmitate-injured human umbilical vein endothelial cells (HUVECs) via modulating reactive oxygen species (ROS)-related inflammation and insulin receptor substrate-1 (IRS-1) signaling [97]. Glucose transporter protein 4 (GLUT4) is the major glucose transporter, whose decrease is one of the important molecular bases of insulin resistance [98]. Recently, it was proven that tectorigenin targeted protein kinase A catalytic subunit α (PKACα) to promote the PKA/AMPK/myocyte enhancer factor 2 (MEF2) pathway, subsequently enhancing GLUT4 expression, and thus slowing and stopping insulin resistance progression for the intervention and treatment of glucose metabolism syndrome [99].

Diabetes is closely linked to the epidemic of obesity [100]. The anti-obesity effects of tectorigenin were also investigated, as evidenced by the decreased body weight, triglycerides, total cholesterol, and low-density lipoprotein cholesterol (LDL-C) in several animal models [42,90,92]. According to the findings of Li et al. [101], tectorigenin restrained 3T3-L1 adipogenesis and reversed TNF-α-induced changes of IL-6, monocyte chemoattractant protein-1 (MCP-1), and adiponectin. Further investigation identified tectorigenin as a functional peroxisome proliferator-activated receptors γ (PPARγ) partial agonist (IC_50_ = 13.3 mM) and suggested that tectorigenin may ameliorate hyperglycemia through the inhibition of preadipocyte differentiation and adipocytokine secretion [101]. The studies presented above highlight the potential therapeutic value of tectorigenin in diabetes and obesity.

### 4.3. Hepatoprotective Effects

The liver plays a pivotal role in bile synthesis, metabolic function, and the degradation of toxins in the body [102]. Several factors, including oxidative stress, lipid peroxidation, and proinflammatory mediators (chemokines and cytokines), are involved in hepatic diseases [103]. Tectorigenin is a potential hepatoprotective agent that has been evaluated by many researchers. It has been demonstrated that tectorigenin exerted protective functions against carbon tetrachloride (CCl_4_) and tert-butyl hyperoxide-induced liver damage [56,104,105,106]. Tectorigenin significantly inhibited the activities of aspartate aminotransferase (AST) and alanine aminotransferase (ALT), and the protective activity of tectorigenin was higher than that of dimethyl diphenyl bicarboxylate and silybin [104,106]. Several studies showed a regulation in the activities of antioxidative enzymes, lipid peroxides, and cytokines in the tectorigenin-treated group, as evidenced by the superoxide dismutase (SOD), ROS, malondialdehyde (MDA), glutathione (GSH), TNF-α, interleukin (IL)-1β levels, etc., which demonstrated that the hepatoprotective mechanisms of tectorigenin might be related to its antioxidant and anti-inflammatory actions [56,105,107]. Liver damage can lead to hepatic fibrosis, cirrhosis, liver failure, and even liver cancer [108]. It has been revealed that tectorigenin exhibited anti-proliferative and pro-apoptotic activities on hepatic stellate cells and human hepatocellular carcinoma HepG2 cells, and might possess anti-fibrotic and anti-hepatoma potential [84,109]. An in vivo study suggested that tectorigenin significantly prevented fat accumulation, promoted bile acid circulation, and exerted beneficial effects on mice with nonalcoholic fatty liver disease through anti-inflammation and improvement of gut microbial dysbiosis [110]. Another study specifically reported the effects of tectorigenin on cholestatic liver disease, and the results proved that tectorigenin alleviated intrahepatic cholestasis via the activation of peroxisome proliferator-activated receptor gamma (PPARγ) and subsequent NF-κB inhibition and bile salt export pump (BSEP) activation [23]. Additionally, tectorigenin mitigated experimental fulminant hepatic failure via modulating the toll-like receptor 4 (TLR4)/MAPK and TLR4/NF-κB pathways and autophagy [107].

### 4.4. Anti-Inflammatory Effects

Several flavonoids, including tectorigenin, have been reported to inhibit nitric oxide (NO) production, one of the inflammatory mediators [111]. Tectorigenin was also found in multiple studies to inhibit the induction of cyclooxygenase-2 (COX-2) in a dose-dependent manner to restrain the production of prostaglandin E_2_ in inflammatory cells. This may be one of the mechanisms of action by which Belamcandae Rhizoma exert an anti-inflammatory effect [112,113,114,115]. Lipopolysaccharide (LPS), an endotoxin, could upregulate inflammatory cytokines, such as IL-1/6/12, TNF-α, p-MAPK, and p-NF-κB. Those changes above were attenuated by tectorigenin in PC12 cells with spinal cord injury [116], LPS-stimulated BV-2 Microglia [115], and mice with acute lung injury [117]. In the A549: THP-1 co-culture model, A549 cells activated and induced THP-1 cells to secrete IL-6, TNF-α, and pro-inflammatory cytokines. Co-incubation of tectorigenin with A549 cells prevented this induction behavior [34]. An in vivo model of acute inflammation study showed that tectorigenin (60 mg/kg) significantly alleviated carrageenan-induced edema in an inflammatory rat model [118]. Wang et al. [24] (2020) also investigated the anti-inflammatory ability of tectorigenin and explored the underlying mechanism in treating the allergic asthma model of guinea pigs. In that study, tectorigenin (25 mg/kg) efficiently reduced the frequency of cough, inflammatory cell numbers, and the content of pro-inflammatory factors. Further research found that tectorigenin might mitigate pulmonary fibrosis and airway inflammation through the TLR4/NF-κB and transforming growth factor β1 (TGF-β1)/Smad signaling pathways, which was consistent with the conclusion of another study that tectorigenin was suggested to play an anti-inflammatory role by antagonizing the activation of the NF-κB signaling pathway [101]. Tectorigenin protected HaCaT keratinocytes against M5 cytokine-induced abnormal proliferation and inflammatory response via suppression of the TLR4/NF-κB pathway [119]. In addition, tectorigenin also protected against multiple organ damage by alleviating inflammation-related pathways or oxidative stress [107,117,120].

### 4.5. Antioxidant Effects

A loss of balance in biological systems between the production of ROS and antioxidant defense levels can induce oxidative stress, which may result in extensive intracellular damage [121,122]. The harmful effects brought by oxidative stress are mainly on account of the overproduction of oxidants or depleting antioxidant potential [123], which causes cellular damage through the oxidation of protein, lipids, and DNA [124,125]. Oxidative stress participates in the nosogenesis of many illnesses, including several cancers, Alzheimer’s disease, and atherosclerosis [125]. Flavonoids are reported as natural scavengers of free radicals, and this may be due to the relatively strong reduction capacity of their phenol groups, which could form resonance-stabilized anion radicals [126]. Both tectorigenin and tectoridin have been reported to show antioxidant ability in vivo and in vitro [42,56]. In an early study, the evaluation of the antioxidant ability of Belamcandae Rhizoma extract containing tectorigenin showed the results of reducing free-radical 1,1-Diphenyl-2-picrylhydrazyl (DPPH) and transition-metal ions and decreasing linoleic acid peroxidation [51]. Another antioxidant capacity assay of tectorigenin displayed that tectorigenin at 10 μg/mL exerted significant intracellular ROS scavenging activity (63.2 ± 2.3%) and DPPH radical scavenging activity (54.3 ± 2.3%), which was superior to tectoridin [127]. In addition, tectorigenin increased the activities and protein expression of the antioxidants, including catalase (CAT), glutathione peroxidase (GPx), and SOD, which might be related to the restraint of the overproduction of ROS and lipid peroxidation. Therefore, the antioxidant capacity of tectorigenin might originate from two aspects: direct scavenging of oxygen free radicals and an indirect effect on the induction of antioxidative enzymes. In vivo experiments also revealed the antioxidative potentiality of tectorigenin, which showed that tectorigenin restrained CCl_4_ and bromobenzene-induced malondialdehyde formation in the rats [56,128]. Tectorigenin sodium sulfonate, reported by Han et al. [60], maintained and elevated the antioxidant activity of tectorigenin through Fe^3+^/ferricyanide reduction, hydroxyl radical and superoxide anion radical scavenging, DPPH, and lipid peroxidation assays in vitro. Another study evaluated the effects of Belamcandae Rhizoma extract containing tectorigenin on collagen degradation and apoptosis in UV-B-induced HaCaT cells. The extract exhibited good free radical scavenging capability and cytoprotective effects, and mitigated cell apoptosis through diminishing caspase-3 levels and increasing the B-cell lymphoma-2 (Bcl-2)/Bcl-2 associated X (Bax) ratio [26]. Taken together, these findings suggest that tectorigenin has a certain antioxidant capacity.

### 4.6. Antimicrobial Effects

The increasing incidence of microorganisms’ drug resistance makes the search for new antimicrobial agents urgent. In this connection, medicinal plants are promising resources [129]. In 2001, two reports revealed the antimicrobial potential of tectorigenin. Its minimum inhibitory concentration (MIC) against four species of dermatophytes of the genera *Trichophyton* ranged from 3.12–6.25 μg/mL [50], and also inhibited the growth of six *Helicobacter pylori* strains (MIC: 50–100 μg/mL) [130]. In another similar study, tectorigenin from the Belamcandae Rhizoma exerted antibacterial activity against *S. aureus* ATCC 33591, *S. aureus* ATCC 25923, which were strains of methicillin-resistant *Staphylococcus aureus* (MRSA), and three clinical isolates of MRSA with MIC values of 125 μg/mL [17]. Further examination confirmed that the anti-MRSA effect of tectorigenin was achieved by suppressing the adenosine triphosphatase (ATPase) and enhancing the permeability of the cytoplasmic membrane. In a cell experiment for in vitro treatment of *Clostridium perfringens* infection, the biofilm formation rates in the presence of 4, 8, 16, and 32 μg/mL tectorigenin were well below the control in a dose-dependent manner [131]. In addition, tectorigenin noteworthily suppressed the gliding movement, biofilm formation, and adhesion of Caco-2 cells by targeting type IV pilus (TFP) and suppressing TFP-associated genes, although it exhibited little antibacterial activity directly against *Clostridium perfringens*. However, more research is still needed to further verify the antibacterial activity of tectorigenin in cells and animals [131].

### 4.7. Bone-Protective Effects

Common skeletal diseases, such as osteoarthritis, osteoporosis, fractures, etc., greatly affect the quality of life of older adults [132]. The homeostasis of bone metabolism is critical for bone health [133]. Bone homeostasis is sustained by the balance between osteoblast-mediated bone formation and osteoclast-mediated bone resorption [134]. Many factors, including aging, postmenopausal estrogen deficiency, and prolonged immobilization, can lead to the disturbance of bone metabolism and result in bone-related diseases [135]. Some phytoestrogens, isoflavonoids, and plant-derived nonsteroidal compounds possessing estrogen-like activity could function as inhibitors of osteoporosis [136]. The healthy functional food containing tectorigenin effectively reduced bone resorption and promoted bone formation in ovariectomized-induced osteoporosis mice [137]. Ma et al. [138] investigated the therapeutic effects of tectorigenin on osteoporosis, and the results showed that tectorigenin reduced mRNA levels of osteoclast-specific genes, including nuclear factor of activated T cell cytoplasmic 1 (NFATc1), tartrate-resistant acid phosphatase (TRAP), matrix metalloproteinase (MMP)-9 and cathepsin K in RAW264.7 cells, and bone marrow mononuclear cells, and mitigated the bone loss in osteoporosis mice [138]. Another study also showed that tectorigenin regulated bone homeostasis by stimulating osteogenic differentiation and inhibiting osteoclast differentiation through bone morphogenetic protein (BMP) and MAPK pathways [120]. Tectorigenin is also attractive as an anti-osteoarthritis drug due to its anti-inflammatory activities. Tectorigenin observably suppressed the NF-κB P65 pathway and inhibited articular cartilage degeneration and chondrocyte apoptosis [139]. Tendinopathy, as a painful overuse musculoskeletal injury, is extremely common in athletes and middle-aged overweight patients [140]. As an inhibitor of MAPK and NF-κB pathways, tectorigenin has the capability to attenuate the inflammation, apoptosis, and ossification of tendon-derived stem cells in vitro and in vivo, thus effectively improving tendinopathy [141].

### 4.8. Anti-Skin-Damage and Antiallergic Effects

Ultraviolet radiation (UV) can induce skin photoaging and inflammation, and also lead to free radical and ROS accumulation, collagen loss and degradation, epidermal pigmentation, and abnormal cell death [142]. Several reports have uncovered the skin-protective effect potential of tectorigenin. Belamcandae Rhizoma extract containing tectorigenin exhibited good free radical scavenging and cytoprotective activities by increasing antioxidant enzyme expression and improving UV-B-induced collagen degradation and apoptosis [26]. Noh et al. [22] also reported that in human keratinocytes, tectorigenin (1 and 10 µM) exerted anti-skin-damage effects through mitigating UV-B-induced hyperoxidation, collagen degradation, and apoptosis. Recently, Dai et al. [143] designed a study to pick out a new retinoic acid γ receptor (RAR-γ)-selective agonist. Computational screening suggested that tectorigenin was a matching selective RAR-γ agonist. In this study, tectorigenin was validated to be able to inhibit UV-induced release of inflammatory factors, oxidative damage, and MMP production, and also to reverse collagen loss. Further research suggested that tectorigenin exerted its effects mainly via regulating the MAPK/c-Jun N-terminal kinase (JNK)/activating protein-1 (AP-1) pathway. Tretinoin can attenuate skin photoaging and inflammation, but its use is restricted because of the strong skin irritation and teratogenic effect [144]. Therefore, it is necessary to find a new agent with protective effects on the skin. These evidences above suggest that tectorigenin has the potential to be an effective strategy for the treatment of UV-induced skin damage. Moreover, Tamura et al. [145] disclosed tectorigenin and another compound from the extract of Puerariae Flos as inhibitors against the expression of immunoglobulin E (IgE) receptor, the key molecule triggering the allergic reactions, via diminishing the generation of γ-chain subunit. Another study demonstrated that tectorigenin effectively attenuated passive skin allergic reaction and restrained IgE-induced release of β-hexosaminidase from RBL-2H3 cells [146].

### 4.9. Cardioprotective and Cerebroprotective Effects

Cardiovascular and cerebrovascular diseases, including angor pectoris, myocardial infarction, stroke, etc., are the leading causes of death globally [147]. Atherosclerosis is the main cause of cardiovascular and cerebrovascular disease development [148]. Blood vessels severely affected by atherosclerosis are prone to enhance platelet aggregation, resulting in vessel occlusion and ischemia [149]. Tectorigenin was an effective antiplatelet compound with a much better effect than acetylsalicylic acid [150]. Tectorigenin was also reported as a lactate dehydrogenase (LDH) inhibitor from several Chinese medicinal herbs by UF-HPLC-DAD-MS [151]. The protective mechanism of tectorigenin was related to oxidative stress and inflammation in which phosphoinositide 3-kinase (PI3K)/AKT, TLR4/NF-κB, PPARγ/NF-κB pathways were involved [39,152,153]. Chen et al. [39] revealed that tectorigenin effectively prevented HUVECs from H_2_O_2_-induced oxidative stress injury by upregulating the PI3K/AKT pathway. In addition, tectorigenin could alleviate cognitive impairment, hippocampal tissue and myelin damage, and inflammation of chronic cerebral ischemia mice [153]. In vitro, tectorigenin benefited HT-22 cell survival and protected against oxygen-glucose deprivation/reoxygenation (OGD/R) damage. However, overexpressing TLR4, or using PI3K/AKT inhibitor LY294002, PPARγ inhibitor GW9662, or NF-κB activator LPS reversed the protection of tectorigenin, suggesting that PI3K/AKT, TLR4, PPARγ, and NF-κB pathways were crucial in the cardioprotective and cerebroprotective effects of tectorigenin [152,153].

### 4.10. Protective Effects on the Respiratory System

Tectorigenin was regarded as an interventional strategy by inhibiting respiratory dysfunction and death in respiratory diseases. Several studies have illustrated the protective effect of tectorigenin in respiratory disorders, including asthma, airway epithelial injury, and pulmonary fibrosis [24,154,155]. Asthma is a common chronic disease characterized by variable respiratory symptoms and airflow limitation [156]. Tectorigenin significantly diminished the frequency of coughs, the number of inflammatory cells, and the levels of inflammation-related factors in the allergic asthma guinea pigs model [24]. For the clinical treatment of asthma, glucocorticoids are currently the first-line drugs, but can also cause airway epithelial injury [24]. It was demonstrated that tectorigenin regulated migration, invasion, and apoptosis of human airway epithelial cells in the treatment of glucocorticoids. Mechanistically, tectorigenin enhanced miR-222-3p expression and inhibited the MAPK pathway, thus protecting the airway epithelium [154]. Idiopathic pulmonary fibrosis is also a chronic, progressive pulmonary disease characterized by the anomalous accumulation of fibrotic tissue in the lung’s parenchyma [157]. In vitro, tectorigenin prevented the proliferation of pulmonary fibroblasts in rats treated with bleomycin, indicating that tectorigenin has the potential to ameliorate pulmonary fibrosis. Further results revealed that its mechanism was related to the regulation of miR-338* (miR-338-5p) expression [155].

### 4.11. Neuroprotective Effects

In neurodegenerative diseases caused by multiple pathological procedures, neuroprotection is an interventional strategy to delay and even stop neuronal dysfunction and abnormal death [158]. Microglia are the primary immune cells in the central nervous system, and their activation is associated with neurodegeneration and alcoholic toxication [159]. Yuan et al. [40] found that isoflavonoids from Puerariae Flos, which included tectorigenin, had obvious inhibitory effects on the release of NO from microglia activated by LPS (IC_50_ values were 1.3–2.3 μM). From the structure-activity relationships of a number of isoflavonoids with inhibitory activity against microglial activation, the methoxyl group at the 6-position of tectorigenin enhanced the activity. Glioblastoma cell viability was dose-dependently decreased after 24 h exposure to tectorigenin, and 200 μM and 300 μM could block the cell cycle arrest at G0/G1 phase [160]. Another study also confirmed the anti-neuroinflammatory effects of tectorigenin in both LPS-treated BV-2 microglial and mouse models [115]. As a glycoprotein hormone, Erythropoietin (EPO) has neuroprotective function [161,162], but EPO circulating in the blood cannot cross the blood-brain barrier [163]. Tectorigenin was proved to promote the stacking of hypoxia-inducible factor (HIF)-1α to induce EPO gene expression in rat cortical neurons and neuron-like NT2/D1 cells, raising endogenous cerebral EPO levels [164]. Monoamine oxidase B (MAO-B) can produce ROS to directly damage neuronal cells, which is a possible target for the treatment of Alzheimer’s disease [57]. Li et al. [57] isolated tectorigenin from *Pueraria thomsonii*, and the IC_50_ value of tectorigenin against MAO-B was 54.36 μg/mL. In addition, pretreatment with tectorigenin exhibited a protective effect against neuronal damage in PC12 cells. Gong et al. (2017) [165] investigated whether tectorigenin could prevent the neurotoxicity of SH-SY5Y cells and illuminated the potential protection mechanism. In that study, tectorigenin (0.1, 1 and 10 μM) exhibited neuroprotective effect against cytotoxicity and apoptosis induced by MPP+ (1-methyl-4-phenylpyridinium), which may be related to the reduction of oxidative stress and the enhancement of the antioxidant defense activity of tectorigenin [165].

### 4.12. Other Effects

Except for the pharmacological activities mentioned above, tectorigenin also has other biological activities. For instance, tectorigenin was verified as a therapeutic strategy for the treatment of obstructive nephropathy. In mice suffering unilateral ureteral obstruction, tectorigenin significantly decreased the levels of the kidney injury index, including creatinine, blood urea nitrogen, and kidney injury molecule-1 (KIM-1), and alleviated pathological damage and renal interstitial fibrosis [166]. In vitro, tectorigenin treatment inhibited Smad3-mediated ferroptosis and fibrosis [166]. It was also revealed that tectorigenin could induce the release of growth hormone in rat pituitary cells [167].

**Table 2:** Biological and pharmacological effects of tectorigenin.

Pharmacological Effects	Animal/Cell Line	Inducer	Route	Dosage	Duration	Mechanism of Action	Results	Ref.
Anticancer effects (Prostate cancer)	PC-3 and LNCaP cells	/	In vitro	10, 50, 100 μM	24 h	Up-regulated ERβ, decreased the tumor cell proliferation- related gene expression	ERβ, PDEF expression↑; PSA, prostate cancer-specific indicator gene DD3 (PCA3), hTERT, IGF-I receptor expression↓	[69]
Anticancer effects (Prostate cancer)	LNCaP cells	/	In vitro	100 μM	24 h	Regulated the aberrant expression of genes relevant in proliferation, invasion, immortalization, and apoptosis	Prostate-derived Ets factor (PDEF), prostate -specific antigen (PSA), human telomerase reverse transcriptase (hTERT), insulin-like growth factor 1 (IGF-1) receptor expression↓; Telomerase activity↓; inhibited MMP and induced apoptosis in LNCaP cells	[73]
Anticancer effects (Prostate cancer)	RWPE-1, LNCaP and PC-3 cells	/	In vitro	50 μM	24, 72 h	Regulated cell cycle	Induced cycle arrest at G1 phase and p21 (WAF1) or p27kip1 protein expression	[75]
Anticancer effects (Prostate cancer)	RM-1, H22 and MFC cells	/	In vitro	/	24, 48 h	/	Exhibited anti-prostate cancer activity with IC_50_ value of 0.08 μM	[53]
Anticancer effects (Breast cancer)	MCF-7 and T-47D cells	/	In vitro	/	96 h	/	Stimulated MCF-7 and T-47D cell proliferation	[55]
Anticancer effects (Breast cancer)	MDA-MB-231, MCF-7 and hMSC cells	/	In vitro	50, 100, 200 μM	24, 48, 72, 96 h	Downregulated matrix metalloproteinases and AKT/MAPK signaling; upregulated caspase family	Induced apoptosis, G0/G1-phase arrest, migration and invasion; MMP-2, MMP-9, Bcl-2, p-AKT, and MAPK expression↓; Bax, cleaved poly [ADP-ribose] polymerase and cleaved caspase-3 expression↑	[78]
Anticancer effects (Ovarian cancer)	MPSC1^TR^, A2780^TR^ and SKOV3^TR^ cells	/	In vitro	25, 100 μM	48 h	Inactivated AKT/IKK/IκB/NF-κB pathway	Activated caspases-3, -8 and -9; Nuclear translocation of NF-κB and FLICE inhibitory protein (FLIP), X-linked inhibitor of apoptosis protein (XIAP), Bcl-2, Bcl-xL and COX-2 expression↓; phosphorylation of IκB and IKK and the activation of AKT↓	[82]
Anticancer effects (Ovarian cancer)	A2780 and IOSE80PC cells	/	In vitro	1.56–100 μM	48 h	/	IC_50_ against A2780 cells is 48.67 ± 0.31 μM	[41]
Anticancer effects (Lung cancer)	A549 cells	/	In vitro	15, 30, 60, 120, 240 μg/mL	24 h	/	Inhibited A549 cells growth with IC_50_ 221.52 μg/mL	[48]
Anticancer effects (Lung cancer)	LLC, S180 and CPAE cells; C57BL/6 mice	Injection of LLCs (in vivo)	In vitro and in vivo	1, 10, 100 μM (in vitro); 30 mg/kg (in vivo)	3 days (in vitro); once a day for 10 days (in vivo)	/	Inhibited tumor growth	[83]
Anticancer effects (Lung cancer)	A549 and THP-1 cells	/	In vitro	10, 25, 50 μM	24 h	Suppressed lung cancer-induced pro-inflammatory response	TNF-α and IL-6 secretion↓; snail expression↓; E-cadherin↑	[34]
Anticancer effects (Hepatocellular cancer)	HepG2 cells	/	In vitro	2.5, 5, 10, 15, 20, 30, 40 μg/mL	12, 24, 48 h	Induced apoptosis via mitochondrial-mediated pathway	HepG2 cell viability↓; induced the condensation of chromatin and fragmentation of nuclei; ROS, intracellular [Ca^2+^], caspase-9 and -3↑; mitochondrial membrane potential↓	[84]
Anticancer effects (Osteosarcoma)	Saos2 and U2OS OS cells	/	In vitro	100, 200, 400 µM	24, 48, 72 h,	Inhibited the proliferation, migration and invasion	Inhibited migration and invasion; cleaved caspase3↑, MMP-1, MMP-2, and MMP-9↓	[21]
Anticancer effects (Colon cancer)	Caco-2 cells	/	In vitro	20 µM	24 h	NF-κB pathway suppression	Arrested invasion; CXCL-10 expression, p-IκB, p-RelA↓; CXCL-10 promoter activity↓	[85]
Anticancer effects (Leukemia)	HL-60, U-937, HepG2 and SNU-C5 cells	/	In vitro	20, 50, 75 μM	1–4 days	Induced differentiation and apoptosis	Inhibited HL-60, U-937, HepG2 and SNU-C cell growth with IC_50_ values of 22.3, 28, 84, and 62.7 μM, respectively; induced HL-60 cells differentiation, caused apoptotic changes of DNA	[86]
Anticancer effects (Testicular cancer)	TGCT, TCam-2 and NTera-2 cells	/	In vitro	100, 250, 500 μM	48, 72 h	Downregulated stem cell factors	Inhibited proliferation, the stem cell factors NANOG and POU5F1↓	[87]
Anticancer effects (Endometrial cancer)	Ishikawa cells	/	In vitro	0.1 μM	72 h	Genomic aberrations	Induced array genes aberrated	[88]
Anticancer effects	COR-L23, C32, MCF-7, HepG2	/	In vitro	50, 100, 200, 400 µM	48 h	Inhibited S phase and G2/M phase	IC_50_ against COR-L23, C32, MCF-7, HepG2 were 189, 207, 149, 105 µM, respectively; 400 µM increased significantly G2/M phase cells	[54]
Anticancer effects	P388, L1210, SNU C4, A549 and MA104 cells	/	In vitro	/	/	/	IC_50_ against P388, L1210, SNU C4, A549 and MA104 were 0.2, 0.04, 0.03, 0.29, >1 mM, respectively	[90]
Antidiabetic effects	Male SD rats	Streptozotocin	In vivo	5, 10 mg/kg	A week	Antioxidant activity	Serum cholesterol, triglyceride, LDL- and VLDL-cholesterol↓; High-density lipoprotein (HDL)-cholesterol↑; DPPH radical, xanthine oxidase, superoxide anion radical, lipid peroxidation↓	[42]
Antidiabetic effects	Male SD rats	Streptozotocin	In vivo	10 mg/kg	Once per day for 3 days	/	Body weight↑; Serum glucose, cholesterol↓	[90]
Antidiabetic effects	Male SD rats	Streptozotocin	In vivo	100 mg/kg	10 consecutive days	/	Exhibited high aldose reductase inhibitory potency with IC_50_ 1.12 μM; inhibited the sorbitol accumulation in red blood cells, sciatic nerves, and lenses by 87.2%, 75.5%, and 50.5%, respectively	[91]
Antidiabetic effects	INS-1, RIN- m5F, and HEK293T cells; Male C57BL/6J mice	Glucose, palmitic acid (in vitro); high-fat +sucrose diet (in vivo)	In vivo and in vitro	40 μg/mL (in vitro); 10, 20, 40 mg/kg (in vivo)	3 h, 9 h, 24 h (in vitro); once every two days for one month (in vivo)	Enhanced PDX1 expression and protected pancreatic β-cells by activating ERK and reducing ER stress	Improved insulin secretion; weight gain, ROS↓; ameliorated hyperglycemia and glucose intolerance and lipotoxicity and apoptosis	[92]
Antidiabetic effects	Rat lenses	/	In vitro	/	/	/	Aldose reductase inhibition IC_50_ = 1.12 ± 0.08 μM	[47]
Antidiabetic effects	Rat lens	/	In vitro	/	/	/	Aldose reductase inhibition IC_50_ = 6.43 μM	[93]
Antidiabetic effects	HRGECs; Male type 2 diabetic (BKS.C g–m +/+ Lepr ^db^/J, db/db) mice, a model homozygous for the diabetes spontaneous mutation	High-glucose (in vitro)	In vitro and in vivo	5, 10 μM (in vitro); 75 mg/kg (in vivo)	24 h (in vitro); 12 weeks (in vivo)	Restored the reduction of AdipoR1/2, pi-LKB1, pi-AMPKα, PPARα, decreased lipotoxicity, reduced macrophage infiltration and macrophage polarization	Attenuated metabolic disorders and exerted renoprotective effects; improved intrarenal lipid metabolism, endothelial functions, and renal insulin sensitivity	[96]
Antidiabetic effects	HUVECs	Palmitic acid	In vitro	0.1, 1, 10 μM	30 min	Inhibited IKKβ/NF-κB/JNK pathway	ROS, MMP, TNF-α, IL-6↓; protected endothelium-dependent relaxation	[97]
Antidiabetic effects	C2C12 myotubes; Male C57BL/6J mice	Palmitic acid (in vitro); high fat (in vivo)	In vitro and in vivo	10 µg/mL (in vitro); 10, 20, 40 mg/kg or 50, 100, 200 mg/kg (in vivo)	24 h (in vitro); every other day for 30 days (in vivo)	PKACα/AMPK/MEF2 pathway	Improved GLUT4, glucose uptake, and insulin sensitivity; ameliorated insulin resistance and hyperglycemia	[99]
Anti-obesity effects	3T3-L1 cells; Male SD rats	Dexamethasone (in vitro); high-fat diet (in vivo)	In vitro and in vivo	10, 25, 50, 75 μM (in vitro); 50, 100 mg/kg (in vivo)	24 h (in vitro); once daily for 2 weeks (in vivo)	PPARγ and IKK/NF-κB pathway	Inhibited adipocyte differentiation; Triglyceride, glycerol-3-phosphate dehydrogenase, adipogenesis-related genes expression↓; IL-6, MCP-1↓; adiponectin secretion↑; increased glucose uptake and insulin sensitivity	[101]
Hepatoprotective effects	male ICR mice	CCl_4_	In vivo	50, 100 mg/kg	Once	Inhibited the β-glucuronidase activity	Inhibited the levels of serum ALT, AST and LDH by 22.4%, 44.4%, and 58.7%, respectively; MDA, Ca^2+^↓, GSH, GST↑	[104]
Hepatoprotective effects	Male SD rats	CCl_4_, high-fat + cholesterol diet, alcohol	In vivo	7.5, 15, 30 mg/kg	Once per day for 6 weeks	Antioxidant activity	Serum ALT, AST, hyaluronate, laminin, procollagen III N-terminal peptide↓; collagen in the livers↓, serum albumin concentration and ratio of albumin to globulin↑, liver lipid peroxidation↓, liver SOD and GPx↑	[105]
Hepatoprotective effects	HepG2 cells; Male ICR mice	t-BHP (in vitro and in vivo)	In vitro and in vivo	0.01, 0.1, 1, 10 μM (in vitro); 25, 50 mg/kg (in vivo)	2 h (in vitro); once (in vivo)	Inhibited the hepatotoxicity	Inhibited the levels of plasma ALT and AST by 39% and 41%, respectively	[106]
Hepatoprotective effects	RAW 264.7 cells; Male C57BL/6J mice	LPS and D-GalN	In vitro and in vivo	1, 10, 100 μM (in vitro); 12.5, 25, 50 mg/kg	24 h (in vitro); once (in vivo)	TLR4/MAPK, TLR4/NF-κB, decreased inflammatory cytokine levels, promoted autophagy	Serum ALT, AST↓; Ameliorated the histological injury, apoptosis, and the mortality	[107]
Hepatoprotective effects	HSC-T6 cells	/	In vitro	10, 20, 40, 60, 80, 100 μg/mL	12, 24, 48 h	Inhibited proliferation and induced apoptosis	Cell viability↓; ROS, intracellular [Ca^2+^]↑; mitochondrial membrane potential↓; translocation of cytochrome c, caspase-3/9↑	[109]
Hepatoprotective effects	Male C57BL/6N mice	High-fat diet	In vivo	25, 50 mg/kg	Once per day for 6 weeks	LPS/TLR-4/NF-κB/TNF-α pathway, modulated gut microbiota	Ameliorated the obese characteristics; total cholesterol, total triglyceride, HDL-C, LDL-C, LPS, total bile acid, ALT, AST↓; fecal total bile acid↑; ameliorated the histological injury	[110]
Hepatoprotective effects	Primary mouse Kupffer cells; Male C57BL/6J mice	ANIT, DDC diet	In vitro and in vivo	10 μM (in vitro); 75 mg/kg	24 h (in vitro); once per day for 5 days (in vivo)	PPARγ/NF-κB, PPARγ/BSEP, inhibited macrophage activation	Serum AST, ALT, γ-glutamyltransferase, and AP↓; ameliorated the histological injury, cell apoptosis, inflammation, and bile metabolic dysfunction	[23]
Anti-inflammatory effects	Raw 264.7 cells	IFN-γ/LPS	In vitro	50, 100, 200 μM	24 h	Blocking of NF-κB activation	Nitric oxide synthase (iNOS)↓, NO↓, IL-1β↓, COX-2↓, Prostaglandin E2 (PGE2)↓	[114]
Anti-inflammatory effects	PC12 cells	LPS	In vitro	25, 50, 100, 200 μM	24 h	Alleviated apoptosis, inflammation, and activation of NF-κB signaling in SCI cell models via inhibiting IGFBP6	Cell viability↑, cell apoptosis↓, caspase-3/8/9, cleaved caspase-3/8/9; IL-1β, IL-6, TNF-α, IGFBP6, TLR4↓; inactivated IκBα and p65	[116]
Anti-inflammatory effects	Female BALB/c mice	LPS	In vivo	5, 10 mg/kg	6 h	NF-κB P65 pathway	Inflammatory cell numbers↓, lung NF-κB p65 mRNA and protein level↓, myeloperoxidase↓, SOD↑, inhibited LPS-induced neutrophils in the lung	[117]
Anti-inflammatory effects	Swiss mice; Wistar rats	Acetic acid or carrageenan	In vivo	50, 100 mg/kg; 10, 60 mg/kg	/	/	In mice, LD_50_ was 1.78 g/kg, had an analgesic effect; in inflammatory rats, reduced carrageenan-induced edema	[118]
Anti-inflammatory effects	HaCaT cells	M5 cytokines	In vitro	2.5, 5, 10, 20, 40 μM	/	TLR4/NF-κB pathway; promoted autophagy	Promoted autophagy: LC3-II/LC3-I, beclin-1, LC3↑; P62↓; suppressed inflammation: IL-6, IL-1β, TNF-α↓; NOD-like receptor family pyrin domain containing 3 (NLRP3), apoptosis-associated speck-like protein (ASC) and caspase-1↓	[119]
Antioxidant effects	Male SD rats	CCl_4_	In vivo	100 mg/kg	Once a day for 7 days	Prevented lipid peroxidation, antioxidation	IC_50_ of free radical scavenging potency was 275 μM; Liver MDA, SOD, CAT, GPx↓ by 75.6%, 63.8%, and 70.4%, respectively; serum AST, ALT↓ by 47.4% and 39.8%, respectively	[56]
Antioxidant effects	V79-4 cells	H_2_O_2_	In vitro	0.1, 1, 10 μg/mL	25 h	Activated ERK pathway; enhanced antioxidative levels, apoptotic and cycle arrest	Prevented lipid peroxidation: intracellular ROS, DPPH↓; apoptotic cells, cell cycle arrest at G2/M↓; Cellular SOD, GPx, CAT↑; cell viability↑	[127]
Antioxidant effects	Male SD rats	Bromobenzene	In vivo	10 mg/kg	Once a day for 7 days	/	Inhibited the AFB_1_-induced mutagenicity by 90% and MNNG-induced one by 76%; prevented the MDA formation	[128]
Antioxidant effects	/	/	In vitro	50, 100, 150, 200 μM	/	/	Exhibited reductive capability and DPPH scavenging activity, IC_50_ of OH^−^, O_2_^•–^ scavenging activity and inhibition of lipid peroxidation were 87, 46.62, and 23 μg/mL, respectively	[60]
Anti-microbial effects (fungal)	7 strains of fungi and 6 strains of bacteria	/	In vitro	/	72 h	/	Inhibited dermatophytes of the genera *Trichophyton* (MIC: 3.12–6.25 μg/mL) and such yeast-like fungi as *Can dida* and *Saccharomyces* (25–50 μg/mL); inhibited the growth of *P. aeruginosa*, *P. vulgαris*, *M. luteus* and *S. aureus* (50–100 μg/mL)	[50]
Anti-microbial effects (HP)	HP ATCC43504, NCTC11637, NCTC11638, HP82516, HP82548 and HP4	/	In vitro	/	3 days	/	Inhibited HP growth (MIC: 50–100 μg/mL)	[130]
Anti-microbial effects (MRSA)	Methicillin-Resistant *Staphylococcus aureus*	/	In vitro	/	18 h	Anti-MRSA action is related to cytoplasmic membrane permeability and ABC transporter	Inhibited all tested strains (MIC: 125 μg/mL)	[17]
Anti-microbial effects (*Clostridium perfringens*)	Caco-2	/	In vitro	4, 8, 16, 32 μg/mL	96 h	Targeting type IV pilus (TFP) system	Inhibited gliding motility, biofilm formation, and adherence to Caco-2 cells; TFP-encoding genes↓	[131]
Bone-protective effects	Bone marrow mononu clear (BMM) cells, RAW264.7 cells; Female C57BL/6 mice	RANKL (in vitro); Ovariectomized model (in vivo)	In vitro and in vivo	10, 40, 80, 160 μM (in vitro); 1, 10 mg/kg (in vivo)	7 days (in vitro); every 3 days for 6 weeks (in vivo)	NF-κB pathway	Reduced osteoclast differentiation, TRAP; NFATc1, cathepsin K, MMP-9↓; reduced the bone loss of trabecular bone; BMD, BV/TV, Tb.N↑, Tb.Sp↓; improved trabecular numbers, decreased osteoclasts numbers	[138]
Bone-protective effects	PDLCs and BMMs; Female ICR mice	Osteogenic medium (in vitro); RANKL, LPS (in vivo)	In vitro and in vivo	10, 50, 100 μM (in vitro); 3 mg/kg (in vivo)	14 days (in vitro); dosing on days 0 and 3 (in vivo)	BMP and MAPK pathways	ALP, OPN, OCN, Runx2, Osterix, BMP-2, BMP-4, Smad-4↑; TRAP, cathepsin-K, MMP-9↓; stimulated osteogenic differentiation, increased bone regeneration, inhibited osteoclast differentiation, and suppressed inflammatory bone loss	[120]
Bone-protective effects	Primary chondrocytes; Male SD rats	Medial collateral ligament transection and medial meniscal tear on the knee joints	In vitro and in vivo	25, 50, 100, 200, 400 μM (in vitro); 0.75, 1.5 μg/kg (in vivo)	24 h (in vitro); every 5 days for 8 weeks (in vivo)	Prevented articular cartilage degeneration and chondrocyte apoptosis via the NF-κB P65 and Bax/Bcl-2/caspase-3 pathway	Type X collagen, cyclooxigenase-2, MMP-3, and MMP-13 expression↓; Runx1, type II collagen, and aggrecan↑; inhibited apoptosis: p-Bad, caspase-3↑, Bax/Bcl-2 ratio↓, improved osteoarthritic injury	[139]
Bone-protective effects	Tendon-derived stem cells; Male SD rats	TNF-α (in vitro); Achilles tenotomy (in vivo)	In vitro and in vivo	50, 100 µM (in vitro); 100 µM (in vivo)	1 h (in vitro); once a week for 8 weeks (in vivo)	NF-κB and MAPK pathways	MMP-3, MMP-9, MMP-13, iNOS, COX-2, IL-6, IL-10, and collagen I, RUNX-2↓; inhibited apoptosis, senescence, and ossification, and alleviated calcification of tendon	[141]
Anti-skin-damage effects	HaCaT cells	Ultraviolet-B light	In vitro	0.1, 1, 10 µM	24 h	Antioxidant, apoptosis, and collagen degradation	Intracellular ROS↓, GSH level↑, GPx, CAT expression↑; caspase-3, Bcl-2/Bax ratio↓; attenuated collagen degradation	[22]
Anti-skin-damage effects	HaCaT, B16-F10, 293T cells; Kunming mice and New Zealand white rabbits	Ultraviolet	In vitro and in vivo	10, 20, 40, 80, 160, 320 µM	24, 48 h	Apoptosis and pyroptosis pathways; MAPK/JNK/AP-1 pathway	Targeting binding RAR-γ; inhibited UV-induced oxidative damage, inflammatory factor releases and MMP production, reversed the loss of collagen	[143]
Antiallergic effects	HMC-1	/	In vitro	10, 25, 50 µM	72 h	Reduced generation of c-chain subunit	Inhibited the expression of IgE receptor	[145]
Antiallergic effects	RBL-2H3 and RAW 264.7 cells	/	In vitro	/	/	Inhibited release of b-hexosaminidase induced by IgE	Inhibited the passive cutaneous anaphylaxis reaction and inhibited the release of β-hexosaminidase from RBL-2H3 cells induced by IgE	[146]
Cardioprotective effects	Human blood	Arachidonic acid	In vitro	/	/	Competitive antagonism at thromboxane receptors	Inhibited whole blood platelet aggregation	[150]
Cardioprotective and cerebroprotective effects	HUVECs	H_2_O_2_	In vitro	0.1, 0.2, 0.5, 1, 10 μM	24 h	PI3K/AKT	Cell viability↑, LDH↓, SOD↑, GSH-Px↑, MDA↓; Attenuated apoptosis	[39]
Cerebroprotective effects	HT-22 cells	OGD/R injury	In vitro	1, 5, 10, 20 mM	1 h	PI3K/AKT and PPARγ/NF-κB pathways	Cell viability↑; LDH, IL-1β, IL-6, TNF-α, ROS↓; alleviated apoptosis	[152]
Cerebroprotective effects	HT-22 cells; Male C57BL/6 mice	OGD/R injury (in vitro); Bilateral carotid artery stenosis (BCAS) (in vivo)	In vitro and in vivo	1, 10, 50, 100, 200 μM (in vitro); 12.5, 25, 50 mg/kg (in vivo)	24 h (in vitro); once daily for 15 days (in vivo)	Inhibited the TLR4/NF-κB pathway	Alleviated cognitive impairment, hippocampal tissue and myelin damage, and inflammation; Cell viability↑; alleviated apoptosis	[153]
Protective effects on the respiratory system	Male guinea pigs	Ovalbumin	In vivo	10, 25 mg/kg	14 days	TGF-β1/Smad and TLR4/NF-κB pathways	Inhibited pulmonary fibrosis and airway inflammation: the number of coughs, inflammatory cells, TGF-β1, p-Smad2/3/4, VEGFA, TNF-α, TLR4, MyD88, NF-κB, p-IKKβ↓; Smad7↑; inhibited pulmonary fibrosis	[24]
Protective effects on the respiratory system	9HTE cells	Dexamethasone	In vitro	0.1, 0.2,0.5, 1, 10 μM	24 h	Enhanced miR-222-3p expression, and inhibited the MAPK pathway	Improved the cell viability, migration, and invasion, and alleviated apoptosis	[154]
Protective effects on the respiratory system	Pulmonary fibroblasts of male SD rats	Bleomycin	In vitro	10, 100, 500 μM	3 days	Enhanced miR-338* (miR-338-5p) expression, inhibited LPA1 expression	Cell viability↓	[155]
Neuroprotective effects	Primary rat microglia	LPS	In vitro	/	/	/	Showed potency of inhibiting NO release from LPS-activated microglia with IC_50_ values of 9.3 µM	[40]
Neuroprotective effects	GBM-8401 and GBM-8901 cells	/	In vitro	25, 50, 100, 200, 300 µM	24 h	Cell cycle arrest	Glioblastoma cell viability↓; cell cycle arrest at G0/G1 phase; p-retinoblastoma protein, cyclin-dependent kinase 4 (CDK4)↓; p21 expression↑	[160]
Neuroprotective effects	BV-2 cells; Male ICR mice (8 weeks)	LPS	In vitro and in vivo	25, 50, 100 μM (in vitro); 5 or 10 mg/kg (in vivo)	24 h (in vitro); once per day for 5 days (in vivo)	NF-κB/ERK/JNK pathway	Intracellular ROS, NO, PGE2, TNF-α, IL-6↓; Extracellular ERK, JNK, iNOS, COX-2↓; Hippocampus MDA, iNOS↓; Serum TNF-α, IL-6 levels↓; TLR4, MyD88 protein↓	[115]
Neuroprotective effects	NT2/D1 cells	/	In vitro	10, 20, 30 μM	24 h	Upregulation of erythropoietin in neurons	Induced transcription of HIF-1α, reduced degradation of HIF-1α-OH	[164]
Neuroprotective effects	PC12 cells	β-Amyloid protein	In vitro	25, 50, 100 μg/mL	12, 24, 48 h	/	IC_50_ against MAO-B was 54.36 μg/mL; had a protective effect against Aβ_(25–35)_-induced cell damage	[57]
Neuroprotective effects	SH-SY5Y	MPP+	In vitro	0.1, 1, 10 μM	24 h	Oxidative stress, apoptosis	Cell viability↑, caspase-3 activity and cytochrome c expression, Bax and Bcl-2 levels↓; ROS and NADPH oxidase↓; SOD, CAT, GPx↑	[165]
Protective effects on obstructive nephropathy	Primary renal tubular epithelial cells; Male C57BL/6 mice	TGF-β1, erastin/RSL3 (in vitro); Unilateral ureteral obstruction (in vivo)	In vitro and in vivo	20, 40, 60 μM (in vitro); 20 mg/kg (in vivo)	24 h (in vitro); daily for 7 consecutive days (in vivo)	Inhibited Smad3-mediated ferroptosis and fibrosis	Blood urea nitrogen and creatinine, KIM-1↓, alleviated pathological damage and renal interstitial fibrosis	[166]
Effects on growth hormone release	Pituitary cells	/	In vitro	2.5–20 μg/mL	15 min	/	Promoted the release of growth hormone, twice as effective as in the control group	[167]

“↑” means up-regulation; “↓” means down-regulation.

## 5. Toxicity of Tectorigenin

Evidence shows that tectorigenin affected Ca^2+^ homeostasis in HepG2 cells [84], HSC-T6 cells [109], isolated liver parenchymal cells [168], and Madin-Darby Canine Kidney cells [169]. The change in Ca^2+^ concentration may trigger some cellular responses, so caution is needed when using tectorigenin. The results of Cheng et al. [169] showed that Ca^2+^ influx might be evoked by protein kinase C (PKC)-insensitive store-operated Ca^2+^ entry and Ca^2+^ release from the endoplasmic reticulum via phospholipase C (PLC)-associated pathways. Tectorigenin at concentrations between 10–60 μM exerted renal cytotoxicity, and the IC_50_ was approximately 45.5 μM. However, using a cytosolic Ca^2+^ chelating agent did not affect the cytotoxicity of tectorigenin, suggesting that tectorigenin caused cell death in a Ca^2+^-independent manner [169]. In terms of other cell models, tectorigenin with a concentration below 80 μmol/L did not cause significant cytotoxicity on BMM cells or RAW267.4 after administration for 24 h or 48 h, but 160 μmol/L tectorigenin significantly decreased the cell viability of BMM cells and RAW267.4 within 48 h [138]. In another study, 200 μM tectorigenin exhibited cytotoxicity in tendon-derived stem cells within 24 h [141]. In addition to the cytotoxicity studies in vitro, in vivo toxicological studies of tectorigenin were also carried out in some experimental animals by a variety of scholars. An acute toxicity study revealed that the LD_50_ of tectorigenin by intragastric administration was 1.78 g/kg in swiss mice, and no significant differences in body weights, food consumption, or hematological or biochemical parameters were observed at tectorigenin’s doses not exceeding 300 mg/kg during a consecutive 28-day treatment [118]. In another acute toxicity test, tectorigenin at a dose of 5 g/kg/day was orally administrated to mice for 14 days, and it did not exhibit acute toxicity [105]. Similarly, tectorigenin (75 mg/kg/day) orally administrated to C57BL/6J mice for 14 days caused no significant renal or splenic toxicity [23]. In streptozotocin and high-fat diet-induced diabetes and obesity models, SD rats gavaged with 50 and 100 mg/kg/day tectorigenin for 14 days showed no significant side effects, including body weight gain, fluid retention, or cardiac hypertrophy [101]. These studies show that tectorigenin may be toxic above a certain concentration or duration, whereas in most cases, tectorigenin did not exert toxicity during the therapeutic range [120,143]. However, the implications of the toxic effects of tectorigenin for clinical use are unclear, so it is necessary to perform a risk assessment of human exposure to tectorigenin.

## 6. Pharmacokinetics of Tectorigenin

**Figure 4:**
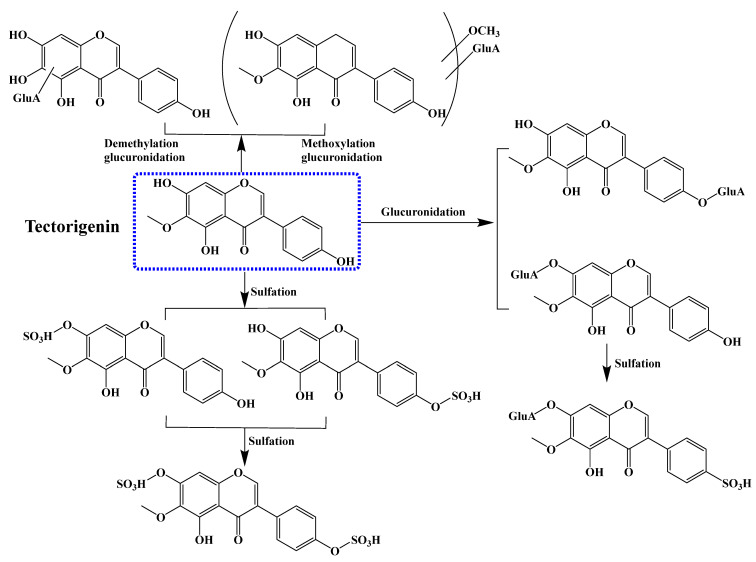
The proposed metabolic pathways of tectorigenin in rats.

The pharmacokinetic properties, including absorption, distribution, metabolism, and excretion, are critical for determining the optimal dose of compounds and the frequency of administration as well as for ensuring drug adherence, and all of these play an important role in clinical application [170]. After rat oral administration, tectorigenin can be directly absorbed from the intestinal tract by passive diffusion, and it then undergoes glucuronidation and/or sulfation metabolic pathways by UDP-glucuronosyltransferases (UGT) and sulfotransferases to form corresponding metabolites [171]. UGT1A1 and UGT1A9 were identified as the primary enzymes catalyzing the glucuronidation of tectorigenin in human liver microsomes [172]. Several investigators have conducted HPLC-MS analyses of metabolites in plasma, urine, and bile after oral tectorigenin consumption in rats. The phase II metabolites of tectorigenin in rat bile and urine were identified by HPLC combined with ion trap tandem and time-of-flight mass spectrometry, and they were either glucuronide and/or sulfate conjugated compounds [173,174]. Shi et al. [175] detected 26 metabolites in rat urine after oral administration of tectorigenin (65 and 130 mg/kg). The results demonstrated that the potential biochemical transformations of tectorigenin included mono-glucuronidation, mono-sulfation, bis-glucuronidation-sulfation, bis-sulfation, bis-glucuronidation, glucuronidation after hydroxylation, methylation, methoxylation, demethylation, etc. [175]. In another study, nine tectorigenin metabolites, including mono-glucuronide and mono-sulfate conjugates, glucuronide-sulfate bis-conjugate, bis-sulfate conjugate, demethylation glucuronide conjugate, and methoxylation glucuronide conjugate, were identified in rats plasma after oral dosing at 65 and 130 mg/kg, and the results of the pharmacokinetic study on six major metabolites showed that the plasma level of mono-glucuronide conjugate tectorigenin-7-O-glucuronide was much higher than tectorigenin and other metabolites [171]. Overall, the main metabolic pathways of tectorigenin in rats include glucuronidation, sulfation, demethylation, and methoxylation, which are displayed in Figure 4.

Nevertheless, due to its low water solubility and low permeability, tectorigenin showed poor absolute bioavailability after oral administration in rats. It was reported that oral tectorigenin (130 mg/kg) administration resulted in a peak concentration (C_max_) of 12.0 ± 0.63 μmol/L, an area under the curve (AUC_0–t_) of 84.2 ± 8.15 μmol/L × h, whereas the long half-life period (t½) 11.7 ± 5.74 h showed that tectorigenin had a long action time [171]. Another study indicated that oral administration of tectorigenin (80 mg/kg) resulted in a C_max_ of 1.46 ± 0.30 μg/mL and AUC_(0–t)_ of 7.31 ± 1.20 μg/mL × h [176]. Additionally, sublingual intravenous administration of tectorigenin (5 mg/kg) resulted in an AUC_(0–t)_ of 219 ± 94 ng/mL × h [177]. Pharmacokinetic studies of some TCMs, such as Shejin-liyan Granule, extracts of Belamcandae Rhizoma and rhizome of *Iris tectorum*, also described the pharmacokinetic characteristics of tectorigenin as the main component [178,179,180]. Tectorigenin was found to exhibit the highest exposure among eight constituents after oral administration of Shejin-liyan Granule [178]. The pharmacokinetic parameters of tectorigenin from multiple studies were summarized in Table 3. Yang et al. [180] found an appearance of a double-peak on the plasma concentration-time curves of tectorigenin after oral administration of *Iris tectorum* extract, and the same phenomenon was observed in some other studies as well [178,179], which suggested that tectorigenin was undergoing enterohepatic circulation, thus resulting in a long excretion time [180]. Moreover, tectorigenin is a metabolite of some isoflavone glycosides and aglycones, including tectoridin [127], kakkalide [181], and irisolidone [182]. Tectorigenin can be quickly generated through the transformation of tectoridin by human intestinal microflora [28,183] and eliminated through urine and feces [184]. However, tectorigenin exerted greater potential than that of tectoridin in many respects [91,185,186]. Therefore, tectoridin may be metabolized into tectorigenin to play a pharmacological role [127]. Although the above research has elucidated some pharmacokinetic characteristics of tectorigenin, more detailed metabolic mechanisms need to be elucidated by conducting more laboratory animal experiments, and due to its low oral bioavailability, more studies should also be carried out to improve the bioavailability.

## 7. Delivery Strategy of Tectorigenin

As described above, the low bioavailability of tectorigenin has severely restricted its clinical application. Generally, many natural flavonoids have the disadvantages of poor water solubility, susceptibility to oxidation, and low bioavailability [187,188,189,190]. These problems can be solved by some methods, such as prodrug design [191], formulation optimization [192], and nano-drug delivery systems, including liposomes [193,194], nanoparticles [195,196], phytosomes [197], dendrimers [198], polymer-drug conjugates [199], and microparticles [200]. Similarly, numerous attempts have been made to increase tectorigenin’s water solubility, stability, and bioavailability by employing diverse delivery strategies. An attempt was made for the first time in 2011 to improve the solubility of tectorigenin by sulfonation, a chemical modification [60]. After sulfonation, the solubility was improved 9-fold and the antioxidant activity increased as well. Solid dispersion is an applicable strategy for the structural transformation of insoluble compounds that have been widely studied to enhance solubility and bioavailability [201]. Shuai et al. [202] prepared the solid dispersions of tectorigenin and the % release was increased 4.35-fold, exceeding pure tectorigenin, and an in vivo experiment proved that the oral bioavailability in rats was also increased. Then, the above team reported that the tectorigenin-loaded self-microemulsifying drug delivery system (TG-SMEDDS) improved physical stability could be kept stable for at least 3 months at room temperature and exhibited good dissolution behavior [203]. An in vivo study proved that TG-SMEDDS had better oral bioavailability both in bile duct ligation and bile duct non-ligation rats. Dai et al. [143] displayed a new drug delivery system that self-assembled to form hyaluronic acid-polyethyleneimine-loaded tectorigenin-nano. When tectorigenin-nano was presented and delivered in the form of nanoparticles, it was better absorbed by skin tissue, improving the bioavailability and exhibiting a better effect than tectorigenin itself [143]. Controlled-release formulations are dosage forms that can release drugs at a regular, quantitative, and uniform rate, and can keep the blood drug concentration for a long time, thereby prolonging the duration of action time and reducing the total dose required for potency [204]. Tectorigenin intragastric floating sustained-release tablets were reported by Wang et al. [205], which could float on the gastric fluid after oral administration, prolong the retention time in the stomach, gradually dissolve, slowly release the drug, and then increase absorption and improve bioavailability.

## 8. Conclusions and Future Perspectives

Tectorigenin, an effective isoflavone, is abundant in a variety of medicinal plants, such as Belamcandae Rhizoma, Puerariae flos, and Chinese water chestnut peel, which are TCMs often used for clearing heat, detoxifying, and diminishing inflammation in many diseases [19,20,48]. Tectorigenin has attracted wide attention as one of the main functional activity ingredients of these TCMs in numerous studies [20,48,114]. This paper reviews comprehensive sources, extraction and synthesis, pharmacology, toxicity, pharmacokinetics, and delivery strategy aspects of tectorigenin in order to provide an important source of information for improving the potential of feasible treatment for a variety of disorders.

The extraction and isolation methods for tectorigenin are relatively simple and mature. Conventional extraction reagents such as methanol, ethanol, and ethyl acetate combined with chromatographic methods can realize the extraction and purification of tectorigenin [40,52,55]. In comparison, the de novo synthesis method of tectorigenin is more complicated. Therefore, extraction from plants with a high tectorigenin content is still the mainstream method to obtain tectorigenin at present. Tectorigenin has multifarious promising medicinal values in anticancer, antioxidant, antimicrobial, anti-inflammatory, and neuroprotective treatments. Through the interpretation and summary of the literature related to tectorigenin, we found that tectorigenin, as well as Chinese herbal extracts containing tectorigenin, exert extensive pharmacological effects by regulating several key enzymes function and the expression of related genes and proteins, including up-regulating antioxidant enzymes such as SOD, GPx and catalase, down-regulating ERβ, IL-6, IL-1β, TNF-α, COX-2, iNOS, and PG-E2, caspase-3/8/9, MMP, p53, and modulating PPARγ/NF-κB, PI3K/AKT, TLR4/NF-κB, IKKβ/NF-κB/JNK, ERK/JNK, MAPK/JNK/AP-1, AKT/MAPK, and TGF-β1/Smad. Existing evidence also suggests that tectorigenin could regulate diverse events involved in cell invasion, immortalization, cell cycle arrest, and cell apoptosis, which obviously supports its traditional application in the treatment of cancer and other diseases. The group and the location of tectorigenin substituents are the key factors for pharmacological activity. Structure-activity relationship analysis of flavonoids suggested that the 5-hydroxyl group and the 6-methoxyl group of the tectorigenin structure are crucial for the inhibitory activity of tumor cells. However, adverse effects of tectorigenin have also been reported, including renal cytotoxicity and hepatotoxicity [84,109,168,169]. The cytotoxicity of tectorigenin is mainly related to the time and concentration of the drug’s administration, whereas in most pharmacodynamic studies, tectorigenin did not exert toxicity during the therapeutic range [23,101,105,120,143]. To ensure the safety of tectorigenin’s clinical application, it is necessary to conduct a risk assessment of human exposure to tectorigenin, carry out more in-depth research on the mechanism of toxicity, and explore ways to reduce its toxicity in the future. In terms of the current pharmacokinetic study, the main metabolic pathways of tectorigenin in rats are glucuronidation, sulfation, demethylation, and methoxylation [171]. However, tectorigenin exhibited poor absolute bioavailability, mainly because of its water-insoluble chemical structure [202]. This may be one of the reasons why fewer experiments are performed on animal models. Meanwhile, due to some differences in pharmacokinetics between the model and normal animals, pharmacokinetic studies should also be carried out on the corresponding model animals.

To advance tectorigenin into a viable clinical therapeutic drug, we summarize several key directions for future research on tectorigenin to conclude this study. (1) Although various biological activities of tectorigenin are confirmed in vitro and in vivo, the molecular mechanisms of action are still unclear, especially the binding sites of definitive target proteins, which remain undetermined. Hence, it is of great significance to further explore the mechanism of its biological activity at the molecular level. (2) The safety of a new drug is especially important before it is used in clinical practice, and more systematic studies of dosage should be conducted to balance both its pharmacological effect and its toxicity. It is also necessary to carry out toxicity evaluations of multiple organs. (3) At present, there are few studies on pharmacokinetics, which should be conducted on more animals in the future, such as dogs and monkeys. Different methods of administration are also worth trying. (4) Pro-drug design, formulation optimization, and novel drug delivery systems, such as nanotechnology, including liposomes, nanoparticles, phytosomes, dendrimers, polymer-drug conjugates, and microparticles, could be attempted to increase tectorigenin’s water solubility, stability, and bioavailability. (5) Structural modification is still an efficient and promising way to obtain tectorigenin derivatives with higher pharmacological activity and relatively better bioavailability, while some key active substituent groups need to be retained.

## Figures and Tables

**Table 1:** Plants containing tectorigenin.

No.	Plant	Family	Part	Ref.
1	*Belamcanda chinensis* (L.) DC.	Iridaceae	Rhizomes	[25,26]
2	*Iris spuria* L. (Calizona)	Iridaceae	Rhizomes	[27]
3	*Iris tectorum* Maxim	Iridaceae	Roots and rhizomes	[28]
4	*Iris japonica* Thunb.	Iridaceae	Whole plant	[29,30]
5	*Iris dichotoma* Pall.	Iridaceae	Rhizomes	[31]
6	*Iris germanica* L.	Iridaceae	Rhizomes	[32]
7	*Iris unguicularis* Poiret	Iridaceae	Rhizomes	[33]
8	*Iris loczyi* Kan.	Iridaceae	Whole plant	[33]
9	*Iris kashmiriana* Baker	Iridaceae	Rhizomes	[34]
10	*Iris crocea* Jacq. ex R. C. Foster	Iridaceae	Roots and rhizomes	[35,36]
11	*Iris ensata* Thunb.	Iridaceae	Rhizomes	[36]
12	*Iris germanica* L.	Iridaceae	Underground parts	[37]
13	*Iris hungarica* Waldst. et Kit.	Iridaceae	Rhizomes	[38]
14	*Iris confusa* Sealy	Iridaceae	Underground parts	[37]
15	*Iris pseudacorus* L.	Iridaceae	Underground parts	[37]
16	*Pueraria lobata* (Willd.) Ohwi	Leguminosae	Flowers	[40,41]
17	*Pueraria thomsonii* Benth.	Leguminosae	Flowers	[18,40]
18	*Pueraria thunbergiana* Benth.	Leguminosae	Flowers	[42]
19	*Dalbergia odorifera* T. Chen	Leguminosae	Leaves	[43]
20	*Dalbergia parviflora* Roxb.	Leguminosae	Heartwood	[44]
21	*Euchresta formosana* (Hayata) Ohwi	Leguminosae	Roots	[45]
22	*Codonopsis pilosula* (Franch.) Nannf.	Campanulaceae	Roots	[46]
23	*Morus alba* L.	Moraceae	Velamen and leaves	[46]
24	*Viola hondoensis* W. Becker et H. Boissieu.	Violaceae	Aerial parts	[47]
25	*Eleocharis dulcis* (Burm. f.) Trin. ex Hensch.	Cyperaceae	Peel	[48]

**Table 3:** Pharmacokinetic parameters of tectorigenin in animals.

Inclusion of Drug Components	Dosage and Route	Animal Model	Pharmacokinetic Parameters	Ref.
Tectorigenin	130 mg/kg, oral administration	Male Sprague-Dawley rats	C_max_ = 12.0 ± 0.63 μmol/L, T_max_ = 0.23 ± 0.15 h, t_1/2_ = 11.7 ± 5.74 h, AUC_(0–t)_ = 84.2 ± 8.15 μmol/L × h	[171]
Tectorigenin	80 mg/kg, oral administration	Sprague-Dawley rats	C_max_ = 1.46 ± 0.30 μg/mL, T_max_ = 1.20 ± 0.30 h, AUC_(0–t)_ = 7.31 ± 1.20 μg/mL × h	[176]
Tectoridin	130 mg/kg, oral administration	Sprague-Dawley rats	C_max_ = 1.08 ± 0.25 μg/mL, T_max_ = 7.11 ± 1.10 h, AUC_(0–t)_ = 8.02 ± 1.10 μg/mL × h	[176]
Tectorigenin	5 mg/kg, sublingual intravenous administration	Male mice	C_max_ = 349.0 ± 172.4 ng/mL, t_1/2_ = 2.4 ± 1.5 h, AUC_(0–t)_ = 219 ± 94 ng/mL × h	[177]
Shejin-liyan Granule	2.0 g/kg, oral administration	Sprague-Dawley rats	C_max 1_ = 112.72 ± 60.04 ng/mL, C_max 2_ = 463.67 ± 170.46 ng/mL, T_max 1_ = 0.75 ± 0.35 h, T_max 2_ = 6.00 ± 2.19 h, t_1/2_ = 12.22 ± 2.42 h, AUC_(0–t)_ = 5340.68 ± 1223.89 ng/mL × h	[178]
Belamcandae Rhizoma extract	50 mg/kg, oral administration	Male Sprague-Dawley rats	C_max_ = 1473.2 ± 156.9 ng/mL, T_max_ = 0.5 ± 0.0 h, t_1/2_ = 3.55 ± 0.34 h, AUC_(0–t)_ = 5800.28 ± 658.0 ng/mL × h	[179]
Extract of rhizome of *Iris tectorum*	46 mg/kg, oral administration	Male Sprague-Dawley rats	C_max_ = 740.3 ± 96.3 ng/mL, T_max_ = 0.2 ± 0.1 h, t_1/2_ = 6.6 ± 2.6 h, AUC_(0–t)_ = 4189.5 ± 60.1 ng/mL × h	[180]
Tectoridin	32 mg/kg, oral administration	Male Sprague-Dawley rats	C_max_ = 476.0 ± 57.8 ng/mL, T_max_ = 0.25 ± 0.2 h, t_1/2_ = 3.4 ± 1.4 h, AUC_(0–t)_ = 1760.9 ± 64.2 ng/mL × h	[180]
Irisolidone	100 mg/kg, oral administration	Male Sprague-Dawley rats	C_max_ = 0.918 ± 0.400 μmol/L, T_max_ = 11.3 ± 1.03 h, t_1/2_ = 12.8 ± 8.28 h, AUC_(0–t)_ = 11.8 ± 5.70 μmol/L × h	[182]
Tectoridin	200 mg/kg, oral administration	Male Sprague-Dawley rats	C_max_ = 8.67 ± 3.07 μmol/L, T_max_ = 4.92 ± 2.87 h, AUC_(0–t)_ = 72.0 ± 22.0 μmol/L × h	[183]

## Data Availability

All data are contained within the manuscript.
